# metaSpectraST: an unsupervised and database-independent analysis workflow for metaproteomic MS/MS data using spectrum clustering

**DOI:** 10.1186/s40168-023-01602-1

**Published:** 2023-08-07

**Authors:** Chunlin Hao, Joshua E. Elias, Patrick K. H. Lee, Henry Lam

**Affiliations:** 1 Department of Chemical and Biological Engineering, The Hong Kong University of Science and Technology, Hong Kong SAR, China; 2 School of Energy and Environment, City University of Hong Kong, Hong Kong SAR, China; 3 Chan Zuckerberg Biohub, Stanford, CA USA; 4 State Key Laboratory of Marine Pollution, City University of Hong Kong, Hong Kong SAR, China

**Keywords:** Metaproteomics, Spectrum clustering, Unsupervised analysis, Gut microbiome

## Abstract

**Background:**

The high diversity and complexity of the microbial community make it a formidable challenge to identify and quantify the large number of proteins expressed in the community. Conventional metaproteomics approaches largely rely on accurate identification of the MS/MS spectra to their corresponding short peptides in the digested samples, followed by protein inference and subsequent taxonomic and functional analysis of the detected proteins. These approaches are dependent on the availability of protein sequence databases derived either from sample-specific metagenomic data or from public repositories. Due to the incompleteness and imperfections of these protein sequence databases, and the preponderance of homologous proteins expressed by different bacterial species in the community, this computational process of peptide identification and protein inference is challenging and error-prone, which hinders the comparison of metaproteomes across multiple samples.

**Results:**

We developed metaSpectraST, an unsupervised and database-independent metaproteomics workflow, which quantitatively profiles and compares metaproteomics samples by clustering experimentally observed MS/MS spectra based on their spectral similarity. We applied metaSpectraST to fecal samples collected from littermates of two different mother mice right after weaning. Quantitative proteome profiles of the microbial communities of different mice were obtained without any peptide-spectrum identification and used to evaluate the overall similarity between samples and highlight any differentiating markers. Compared to the conventional database-dependent metaproteomics analysis, metaSpectraST is more successful in classifying the samples and detecting the subtle microbiome changes of mouse gut microbiomes post-weaning. metaSpectraST could also be used as a tool to select the suitable biological replicates from samples with wide inter-individual variation.

**Conclusions:**

metaSpectraST enables rapid profiling of metaproteomic samples quantitatively, without the need for constructing the protein sequence database or identification of the MS/MS spectra. It maximally preserves information contained in the experimental MS/MS spectra by clustering all of them first and thus is able to better profile the complex microbial communities and highlight their functional changes, as compared with conventional approaches. tag the videobyte in this section as ESM4

Video Abstract

**Supplementary Information:**

The online version contains supplementary material available at 10.1186/s40168-023-01602-1.

## Background

Over the past few years, metaproteomics has become an invaluable technology for directly characterizing the functional roles of microbial communities and associating them with the corresponding host phenotypes, complementing the information offered by metagenomics and metatranscriptomics [1]. However, due to the high diversity and complexity of microbial communities (gut microbiome, for example), it remains a formidable challenge to identify and quantify the large number of proteins expressed in a community, let alone comparing across multiple samples. Identifying and quantifying proteins by mass spectrometry-based proteomics largely rely on the pre-curated protein sequence databases, against which the observed MS/MS spectra are searched. Ideally, such a search database should include all of the protein sequences that cover the whole genetic potential of the microbial community. However, many organisms in microbial communities lack complete, annotated genomes [2, 3]. As an alternative, publicly available gene or protein databases, such as NCBI RefSeq, Ensembl, and Uniprot, can be compiled and used for searching. Unfortunately, although these public databases are growing rapidly, they are far from complete with many species and their protein sequences still missing [4]. Moreover, without any a priori knowledge of the taxonomic composition of the samples of interest, one would like to include protein sequences of as many species as possible, but doing so results in an excessively large search database with high redundancy. Searching the large and redundant database is computationally intensive and complicates the peptide-spectrum matching and subsequent statistical validation in proteomic data analysis and generally leads to fewer peptide/protein identifications [2, 5, 6].

To address this problem, researchers have developed a variety of dedicated analytical methods for metaproteomics analysis. Many of these methods, such as MetaLab [7], MetaPro-IQ [8], and ProteoStorm [9], adopt an iterative search strategy, in which the search database undergoes sequential refinement through multiple rounds of searches, with each round of search providing information to create a smaller database for the next round of search. Such iterative search strategies are capable of handling large and redundant search databases and are shown to substantially increase the number of peptide/protein identifications [10]. However, iterative search could potentially underestimate the false discovery rate (FDR) and result in false-positive identification of proteins from species that is not even present in a sample [2]. On the other hand, with continuous advances in metagenomic sequencing and genome assembly techniques, sample-specific protein sequence databases can be derived from the metagenome-assembled genomes (MAGs) recovered from microbial communities. Compared with public databases, the sample-specific search database has a lower level of redundancy and ambiguity and is much smaller in size. Therefore, it outperforms public databases in terms of the number of identifications and simplifies downstream processing [6]. But building such a sample-specific database requires extra experiments, which implies longer studies and higher cost. At the same time, the errors in genome assembly from sequencing reads and gene prediction will be propagated to peptide-spectrum matching in metaproteomics [11]. No matter how the sequence database is constructed, metaproteomic experiments tend to have a lower rate of identification than in single-organism proteomics, with a large fraction of spectra not confidently identified, likely due to the imperfections of the search database. This results in a substantial information loss that makes it even harder to conduct meaningful biological experiments on microbial communities.

Protein inference is another challenge in metaproteomics, which affects both sample-specific and public search databases. Observation of peptides that are unique to a single protein can be taken as evidence for the existence of that protein, which is often possible in single-organism proteomics. However, one cannot make such inference for peptides whose sequence are commonly shared by multiple homologous proteins beyond the fact that one or more of those proteins should be in the sample. In the context of microbial communities, the presence of closely related species or conserved sequences across species will cause most peptides to be shared among many proteins [12]. In single-organism proteomics, homologous proteins that cannot be confidently resolved are pooled as a “protein group”. In metaproteomics, however, a protein group can contain hundreds of different proteins due to shared peptides, and such grouping of similar proteins may differ between different samples, making it impossible to perform differential protein abundance analysis across samples [13]. The conservative approach of neglecting all shared peptides, which is sometimes practiced in single-organism proteomics, would imply throwing away most of the data in metaproteomics. Therefore, instead of assembling the protein groups based only on the detected shared peptides after searching, a better approach is to apply multiple sequence alignment on whole protein sequences and group homologous proteins as one functional “pan-protein” unit before searching, with the assumption that proteins which share significant sequence similarity may have very similar functional roles [14–16]. By doing so, one does not attempt to connect the expressed function to the species that is/are responsible for the function, but this kind of taxon-agnostic functional analysis nonetheless enables one to make the most out of metaproteomics data, given current technological limitations.

In this study, we propose an unsupervised and database-independent analysis workflow for metaproteomic MS/MS data, referred to as metaSpectraST, which bypasses the peptide/protein identification step and performs proteome comparison between samples solely on the MS/MS spectra acquired. The cornerstone of this workflow is to cluster all experimentally observed MS/MS spectra based on their spectral similarity and create a representative consensus spectrum for each spectrum cluster by using the spectrum clustering algorithm implemented in SpectraST, a spectral library search engine widely used in proteomics [17]. Spectrally similar MS/MS spectra that are grouped in a cluster are presumed to stem from the same peptide sequence [17–20]; that is, they are replicates of the same peptide either from the same or different samples. Thus, for a metaproteomic sample, we can easily obtain its community profile by counting the number or signal intensity of the constituent replicate spectra of each spectrum cluster in the sample. Since the consensus spectra are created by taking all samples of interest into consideration, the entire set of consensus spectra becomes a unified basis for comparing across samples. A second advantage of this community profile is that it maximally preserves information of a microbial community, as spectra that cannot be assigned to any peptide sequence via database search are also retained. This advantage makes metaSpectraST capable of detecting subtle differences between samples, which is useful when there is wide inter-individual heterogeneity of samples. In addition, consensus spectra are often of higher quality in terms of signal-to-noise ratio and mass accuracy than their constituent replicate spectra and thereby have a higher chance to be confidently identified in principle [21, 22]. By analyzing the consensus spectrum and its constituent replicate spectra as a whole, and making the reasonable assumption that they should be identified to the same peptide, one can readily correct search engine errors by comparing and reconciling the identifications of spectra within a spectrum cluster.

We applied the metaSpectraST workflow on the gut microbiomes of 16 mice and compared the results with conventional metaproteomic analysis using MAGs-derived sample-specific databases. We demonstrated that metaSpectraST can better characterize the subtle features of microbial communities, resulting in better classification of samples. We also employed various identification methods, including database search, open modification search, and de novo sequencing, to identify the consensus spectra and their constituent replicate spectra, and developed a reconciliation scheme to determine a consensus peptide sequence for each of the spectrum cluster.

## Methods

### Mouse breeding and experimental setup

**Fig. 1:**
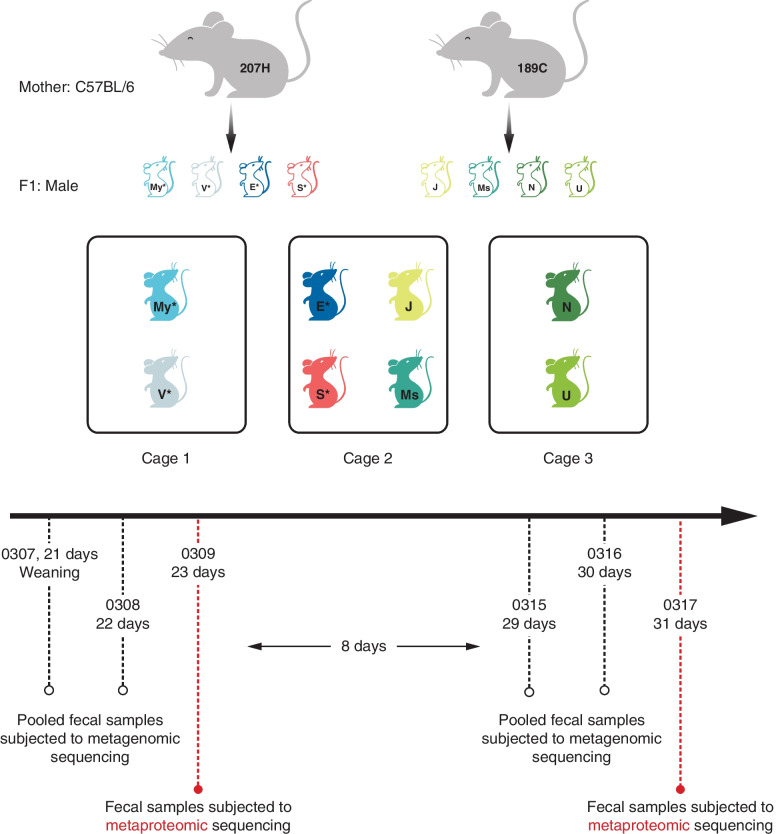
Overview of experimental design. Eight male C57BL/6 mice from two mother mice were housed in three different cages. Littermates, My^*^ and V^*^, and N and U were co-housed in cage 1 and cage 3, respectively, while littermates E^*^ and S^*^ were co-housed together with littermates J and Ms in cage 2. Sample name with and without asterisk indicates littermates of mother mouse 207H and 189C, respectively. Fecal samples of each mouse were collected on the 1st, 2nd, 3rd, 9th, 10th, and 11th day after weaning. Samples from the 1st and 2nd day as well as the 9th and 10th day after weaning were respectively pooled for metagenomic sequencing. Samples from the 3rd and 11th day were subjected to metaproteomic analysis separately

Eight male C57BL/6 mice were used in this study. Four (denoted by My^*^, V^*^, E^*^, S^*^) of the mice were from the same litter of one biological mother (207H), and the other four mice (denoted by J, Ms, N, U) were from the same litter of another biological mother (189C). To explore the potential maternal and co-housing effects, the eight mice were housed in three different individual ventilated cages as follows: My^*^ and V^*^ (littermates of mother mouse 207H) were housed in cage 1; N and U (littermates of mother mouse 189C) were housed in cage 3; E^*^ and S^*^ (littermates of mother mouse 207H); and J and Ms (littermates of mother mouse 189C) were co-housed in cage 2 (Fig. 1). All mice were housed in a 12-h light/dark cycle and fed irradiated water and standard food after weaning at the age of 21 days. Mice were obtained from the Animal and Plant Care Facility of The Hong Kong University of Science and Technology and were bred at the core facility. All experimental procedures involving animals were conducted in compliance with the Animal User Manual and approval was obtained from the Animal Ethics Committee of The Hong Kong University of Science and Technology.

### Fecal sample collection

Fecal samples of each mouse were independently collected on the 21st, 22nd, 23rd, 29th, 30th, and 31st day after birth. Fresh fecal samples were weighted and immediately frozen and kept at −80 $^{\circ }$C. For individual mouse, fecal samples from the 21st and 22nd day ($\sim$ 3 weeks of age, 1st and 2nd day after weaning), and samples from the 29th and 30th day ($\sim$ 4 weeks of age, 9th and 10th day after weaning), were pooled and subjected to metagenomic sequencing, respectively; samples from the 23rd day ($\sim$ 3 weeks of age, 3rd day after weaning) and the 31 days ($\sim$ 4 weeks of age, 11th day after weaning) were subjected to metaproteomic analysis separately (Fig. 1).

### DNA extraction and shotgun metagenomic sequencing

DNA was extracted and purified following the standard method described by Qin J. and colleagues [23]. Paired-end sequencing library was constructed for each of the pooled fecal samples and sequenced by the BGISEQ-500 platform according to the manufacturer’s instruction. In total, 16 samples were sequenced. Each mouse had two sets of metagenomic data, which represented its gut microbiome on the 1st and 10th day after weaning, respectively.

### Short-read de novo assembly and contigs binning

Adaptor sequences and low-quality reads were filtered out by SOAPnuke (v1.5.6) [24] with the following settings: “-l 20 -q 0.2 -n 0.05 -Q 2 -d -c 0 -5 0 -7 1.” Host reads were removed by aligning against the *Mus musculus* complete genome using Bowtie2 (v2.2.5) [17]. The resulting clean reads of each sample were assembled independently by MEGAHIT (v1.1.3) [25] with the following settings: “--min-count 2 --k-min 33 --k-max 83 --k-step 10.” For each sample, the assembled contigs were binned into metagenome-assembled genomes (MAGs) independently with three different methods, CONCOCT [26], MaxBin 2 [27], and MetaBAT 2 [28], using the default settings. The three sets of MAGs produced by different binning algorithms were then dereplicated and refined by considering their quality (i.e., contamination, completeness, and assembly N50) with DAS tool [29], at a $S_{b}$ threshold of 0.5 (weighting factors $b=0.6$, $c=0.5$). The refined MAGs from all samples were pooled and dereplicated again to create a unique set of MAGs using dRep [30]; the minimum average nucleotide identity (ANI) for primary and secondary clusters were respectively 90% and 99%, and the minimum aligned fraction was 10%. The quality of the unique set of MAGs were assessed by CheckM (v1.1.3) [31, 32] (Supplementary Fig. S2).

### Phylogenetic analysis of MAGs

The taxonomy of each MAGs was assigned using the classify_bins module of metaWRAP (v1.2) [33]. And the circular representation of the phylogenetic tree of MAGs was produced by GraPhlAn (1.1.3) [34].

### Estimation of the relative abundance of MAGs

The clean reads from each data set were mapped to the MAGs using Salmon (1.5.2) [35] with the option “--validateMappings.” The relative abundance of each MAG was determined by the total number of reads mapped to the MAG divided by the MAG size and then was normalized by the TMM (trimmed mean of M values) method across samples [36].

### Rarefaction analysis

Taxonomic classification of short reads was performed using Kraken 2 and its standard database [37]. The rarefied species richness was then estimated by the R package Vegan (2.5-7) [38].

### Gene prediction and construction of unique gene set

Open reading frames (ORFs) were predicted from the assembled contigs of all samples by MetaGeneMark (v2.10) [39] with the default settings. Predicted ORFs were clustered at 95% nucleotide identity over 90% of the length of the shortest sequence to create a set of unique genes of all samples, using CD-HIT (v4.6.6) [40].

### Gene functional analysis

Functional analysis of genes in the unique gene set was performed by translating and mapping the nucleotide sequences against the NCBI nonredundant Protein Sequence Database (v20180814, microorganisms only) using the “blastx” function of DIAMOND (v0.8.23.85) [41] with the following settings: “--id 90 --evalue 1e-5 -k 1 --max-hsps 1.” eggNOG-mapper (v2) [42] was used to retrieve the KEGG orthology (KO) terms and pathways of the predicted genes from the Kyoto Encyclopedia of Genes and Genomes (KEGG) database (http://geneontology.org/).

### Protein sequence database and protein clustering

The sample-specific protein sequence database for database search of MS/MS spectra was created by translating the nucleotide sequences in the unique gene set to amino acid sequences.

The all-vs-all BLASTP [43] was then performed on the sample-specific protein sequence database with the setting “-evalue 1.0e-05.” Homologous proteins, which often have the same or closely related function, were then clustered into MCL-clustered protein groups using the Markov Cluster Algorithm (MCL) [44] with an inflation value of 1.5 [16], regardless of their corresponding taxons. Since homologous proteins across different species are presumed to have the same or closely related function, each MCL-clustered protein group is given a functional annotation that matches the majority of proteins in the cluster.

### Metaproteomic sample preparation

Fecal samples (30–50 mg) were lysed in SDS lysis buffer (100 $\upmu$L, 4% SDS, 50 mM Tris-HCl pH 8.2, $1 \times$ cOmplete EDTA-free protease inhibitor) and disrupted with 2 g of 2.3 mm and 0.3 g of 0.1 mm Biospec zirconia/silica beads, followed by ultrasonication in cold water for 10–15 min using the OMNI SONIC RUPTOR 400 with 90% amplitude. The lysate was further incubated at 95 $^{\circ }$C and 600 rpm for 10 min. Beads and any insoluble material was removed by centrifugation at 16,000 RCF and 25 $^{\circ }$C for 20 min. Proteins were then precipitated by 4 times the sample volume of cold acetone at −20 $^{\circ }$C overnight. Proteins were spun down by centrifugation at 16,000 RCF and 0 $^{\circ }$C for 40 min and washed with cold (−20 $^{\circ }$C) washing buffer (mixture of 80% acetone and 20% of a methanol/H2O/acetic acid [50:49:1, v/v/v] solution) to remove any impurities. The protein pellets were resuspended in reconstitution buffer (6 m urea, 50 mM (NH$_{4}$)HCO$_{43}$, and 600 mM guanidine HCl). Protein concentration was determined by the Bicinchoninic acid assay (BCA) following the manufacturer’s instructions (Pierce BCA Protein Assay Kit). An aliquot of 50 $\upmu$g of dissolved proteins of each sample was used for the subsequent reduction, alkylation, and tryptic digestion. Briefly, proteins were reduced and alkylated by 10 mM dithiothreitol (DTT) and 20 mM 2-iodoacetamide (IAA), respectively, and then digested at 37 °C overnight by sequencing grade modified trypsin ($W_{t} : W_{p} = 1 : 50$). The tryptic digest was desalted using C18 Spin Tips (Thermo Fisher Scientific). The desalted peptide mixture was vacuum centrifuged to dryness and suspended in 10 $\upmu$L of 0.1% (v/v) formic acid for LC-MS/MS analysis.

### LC-MS/MS

Metaproteomic samples were randomly analyzed on the Q Exactive HF-X hybrid quadrupole-Orbitrap mass spectrometer coupled with the Easy-nLC 1000 system (Thermo Fisher Scientific). An in-house laser-pulled 75 $\upmu$m i.d. $\times 200$ mm column with integrated spray tip and packed with 1.9 $\upmu$m, 120 Å ReproSil-Pur C18 resins (Dr. Maisch GmbH) was used.

Two $\upmu$g of peptides of each sample was separated and eluted by the mobile phase composed of A = 0.1% formic acid in water and B = 0.1% formic acid in acetonitrile at a flow rate of 250 nL/min over a 80-min gradient (3–7% B 2 min, 7–22% 50 min, 22–35% 10 min, 35–90% 2 min, 90% 16 min). Eluant was ionized by the electrospray ionization (ESI) method, followed by a full MS scan from 300 to 1500 m/z in the Orbitrap mass analyzer at a mass resolution of 60,000. The automatic gain control (AGC) target and the maximum injection time (IT) for full MS scan was set to 2.0e5 and 100 ms, respectively. The MS/MS scan was performed in the TOP 20 data-dependent mode, at a mass resolution of 15,000. The precursor ions were selected by the quadrupole mass analyzer with an isolation window of 1.6 m/z and a dynamic exclusion duration of 30 sec, followed by the high-energy collision dissociation (HCD) fragmentation with a normalized collision energy (NCE) of 27%. The automatic gain control (AGC) target and the maximum injection time (IT) for MS/MS scan was set to 5.0e4 and 45 ms, respectively.

### MS/MS spectrum clustering and creation of consensus spectra

The mass spectrometry data was acquired in the format of RAW and converted to the mzML format by MSConvert of ProteoWizard (v2.1x) [45] with the default settings. A total of 16 MS/MS data sets in mzML format were imported into SpectraST (v5.0) [17, 19] with fragmentation tag “HCD.” Low-quality spectra that were not likely to be peptide spectra were removed. All MS/MS spectra were then clustered based solely on spectral similarity without any knowledge of their corresponding peptide sequences, and replicate spectra (experimental MS/MS spectra clustered together) were combined to create a consensus spectrum. Details of the spectrum clustering algorithm and creation of consensus spectrum were described previously by Lam et al. [17] and Önder et al. [19]. To further demonstrate the effectiveness of metaSpectraST in another metaproteomics data set, an Arctic ocean microbiome dataset was downloaded from ProteomeXchange (PXD008780) and run through the exact same data processing pipeline. In brief, the data set consists of 26 RAW mass spectral files of microbiome samples collected from Bering Strait and Chukchi Sea. Samples then went through a 10-day shipboard incubation with or without organic material input to simulate the effects on ocean microbiome of algal bloom and oligotrophic control, respectively.

### Quantitative profile of microbial community

The microbial community of each metaproteomic sample was quantitatively profiled by counting the number or signal intensity of the constituent replicate spectra of each consensus spectrum created in spectrum clustering step. The number of constituent replicate spectra of a particular consensus spectrum in each sample, referred to as spectral count (SC) of that consensus spectrum, was normalized by the sum of SC of that sample. The spectral index (*SI*) method proposed by Griffin et al. [46] was also adapted to quantify the relative abundance of the contributing peptide ion in each sample based on the intensity profiles of the constituent replicate spectra of a certain consensus spectrum. As originally described, the *SI* of a protein was the sum of fragment ion intensity of all primary fragments (b and y ions) of all MS/MS spectra that are identified to peptides mapped to that protein, and it was then normalized by the protein length and the total *SI* of the data set, known as normalized spectral index ($SI_{N}$). Here, primary fragments cannot be identified from the MS/MS spectra without peptide identifications. Instead, the *SI* of a consensus spectrum was calculated as the cumulative fragment ion intensity of peaks in its constituent replicate spectra that can be aligned with peaks in itself, and was defined as

$$\begin{aligned} SI = \sum \limits _{k=1}^{rn}\left( \sum \limits _{j=1}^{pn}I_{j}\right) _{k} \end{aligned}$$

where *pn* was the number of aligned peaks for constituent replicate spectrum *k*, *I* was the ion intensity of peak *j*, and *rn* was the number of constituent replicate spectra. The tolerance window of alignment was set to $\pm 0.4$ Th. *SI* of the consensus spectrum was normalized by a pseudo length, which was calculated as the molecular weight (MW) of the consensus spectrum divided by the weighted average amino acid residue mass (110 Da):

$$\begin{aligned} SI_{N} = \sum \limits _{k=1}^{rn}\left( \sum \limits _{j=1}^{pn}I_{j}\right) _{k} /\left( \frac{MW}{110}\right) \end{aligned}$$

Finally, $SI_{N}$ of all consensus spectra was normalized across all samples using the TMM method (trimmed mean of M values) [36]. Note that the $SI_{N}$ here is a measure of the abundance of a putative peptide, not of a protein as in the original spectral index method.

### Unsupervised hierarchical clustering

Metaproteomic and metagenomic samples were hierarchically clustered based on consensus spectra $SI_{N}$ (or *SC*) and MAGs abundance, respectively, using the Euclidean distance metric and average linkage criterion. The data matrix of consensus spectra $SI_{N}$, *SC*, or MAGs abundance was augmented by adding the minimum value of the matrix to impute the missing values, and then was *log*2 transformed as the input of unsupervised hierarchical clustering.

### Database search

Database search of both consensus spectra and experimental MS/MS spectra (mzML format) was performed by Comet (2019.01 rev. 5) [47]. Search parameters were set as follows: peptide mass tolerance = 20.00 ppm; mass type parent = monoisotopic masses; fragment bin tolerance = 0.02; fragment bin offset = 0.0; mass type fragment = monoisotopic masses; search enzyme = trypsin; the number of enzyme termini = fully digested; allowed missed cleavage = 2. The oxidation of methionine ($\Delta m$ = 15.9949 Da) was set as variable modification, and the carbamidomethylation of cysteine ($\Delta m$ = 57.021464 Da) was set as additional modification. The maximum variable modifications per peptide was 5. Comet search results were statistically validated using PeptideProphet [48], iProphet [49], and ProteinProphet [50]. The iProphet and ProteinProphet estimated false discovery rate (FDR) were both set to 0.01. The metagenome-derived protein sequence database, constructed as described above, was searched to identified peptides/proteins expressed in the microbial communities. The *Mus musculus* reference proteome (UP000000589, containing 53106 proteins, downloaded from UniProt on 6 Apr 2018) was searched to identify peptides/proteins expressed by the host. The search database was appended with an equal-size decoy sequence database by the decoyFastaGenerator function of the Trans-Proteomics Pipeline (TPP) (v5.1.0). Protein groups identified through the database search were subsequently quantified by StPeter (v1.2.4) [51] (measured as $SI_{N}$) with degenerate peptides option on. The StPeter mass tolerance for matching MS2 peaks was set to 0.4 Da.

### Open modification search

Open modification search of the experimental MS/MS spectra was performed against the UniProt bacterial protein sequences database (SwissProt and TrEMBL, downloaded on 26 July 2018) by TagGraph (v1.7.0.1) [52]. The expected standard deviation of the fragment mass error distribution was 10 ppm. The mass tolerance of a candidate modification was 0.1 Da. The maximum number of occurrences of a de novo-produced substring in the protein sequence database was 5000 and 1000 when that substring was considered as an unmodified and modified peptide match, respectively. The number of iterations in the initial expectation maximization (EM) was 20. The maximum number of EM iterations for FDR assignment was set to 100.

### De novo sequencing

The commercial software PEAKS studio X+ was used for the de novo sequencing of the experimental MS/MS spectra, with the following options: precursor mass range = 300.0–400.0 Da; precursor mass error tolerance = 10 ppm; fragment ion mass error tolerance = 0.05 Da; enzyme = trypsin; PTM = carbamidomethylation, Oxidation (M); maximum allowed variable PTM per peptide = 3; Report candidates per spectrum = 5. The average of local confidence (ALC) threshold was set as 60%.

### Identification reconciliation among the consensus spectrum and its constituent replicate spectra

To maximize the chance of peptide identification, consensus spectra and their constituent replicate spectra were analyzed by multiple identification approaches, including database search, open modification search, and de novo sequencing. A heuristic reconciliation scheme was developed to resolve the conflicting sequences identified by different approaches and determine the consensus peptide sequence (and protein if applicable) of each consensus spectrum. For each spectrum analyzed, the sequence assigned by database search would be preferred whenever a spectrum can be identified by database search; if a spectrum cannot be identified through database search, the sequence given by open modification search would be chosen; the sequence assigned by de novo sequencing would be used if and only if a spectrum failed to be identified by neither database search nor open modification search. Subsequently, a sequence “voting” procedure was adopted to determine the final consensus peptide sequence of each consensus spectrum, whereby the most frequently identified sequence among the consensus spectrum and its constituent replicate spectra would be chosen.

**Fig. 2:**
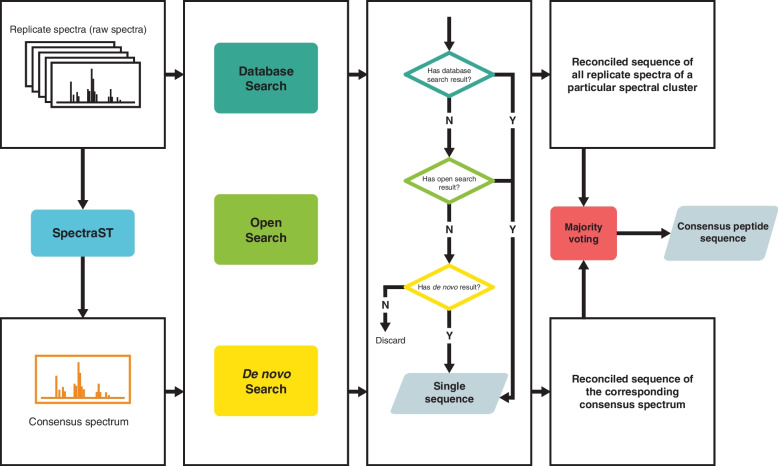
Reconciliation scheme for consensus peptide sequence of each spectrum cluster. Consensus spectrum and its replicate spectra were identified by multiple sequencing methods. For each spectrum (consensus and replicate spectrum), the peptide sequence was determined in a hierarchical order of database search, open modification search, and de novo sequencing. The peptide sequences of consensus spectrum and all of its replicate spectra voted for the final consensus peptide sequence of that spectrum cluster according to the majority rule

The rationale behind this reconciliation scheme is threefold: (1) database search with its relevant statistical validation is the most robust and reliable method in terms of peptide/protein identification, especially when sample-specific protein sequence database is available; (2) open modification search also relies on the reference protein sequence database, albeit a more general one encompassing all bacterial proteins in Uniprot, and should be able to identify closely related sequences that are not in the database due to its allowance of amino acid substitutions; and (3) de novo sequencing is relatively error-prone and often assigns shorter sequences to the spectra, which are less likely to help identify its parent protein in the protein inference process. The voting scheme was designed to correct identification mistakes of spectrum clusters, with the presumption that the correct identification are likely to be repeated among replicate spectra, while incorrect identification tends to hit different sequences stochastically. The consensus spectrum itself contributes an additional “tie-breaking” vote, which typically matters only in cases with very few replicate spectra. The whole reconciliation scheme is illustrated in Fig. 2.

### Statistical analysis

Welch’s ANOVA with bootstrapping ($n=10,000$) was conducted to detect differences in abundance (as inferred by consensus spectrum $SI_{N}$) across multiple sample clusters generated by unsupervised hierarchical clustering. Multiple-testing correction was done by Benjamini-Hochberg procedure, controlling the FDR at 0.05. The following post-hoc analysis was performed using the Games-Howell test with a *p*-value of 0.05.

The differential functional analysis across samples/clusters was conducted on the MCL-clustered protein group basis, where the abundance of MCL-clustered protein group was measured as the sum total of intensity of peptides identified in conventional metaproteomic workflow, or as the sum total of intensity of consensus peptides mapped to the same MCL-clustered protein group. Welch’s *t*-test was then performed to detect differences in MCL-clustered protein groups between sample groups (groups organized by the time of sampling) or sample clusters (as classified by metaSpectraST and principal component analysis), followed by Benjamini-Hochberg multiple-testing correction at an FDR cutoff of 0.05. The post-hoc analysis was performed using the Games-Howell test with a *p*-value of 0.05.

### Enrichment analysis of KEGG pathways

The enrichment analysis of KEGG pathways (level 2) was performed using Fisher’s exact test (two-tailed *p*-value of 0.05), considering all proteins (corresponding to consensus spectra) that were identified by database search as the reference background. Benjamini-Hochberg multiple-testing correction was applied with an FDR cutoff of 0.05. Here, we only considered consensus spectra whose sequences were determined by database search, because open modification search or de novo sequencing cannot indicate the biological function of a consensus spectrum explicitly.

### Code availability

metaSpectraST and the user guides are available at https://github.com/bravokid47/metaSpectraST.

## Results and discussion

### General considerations for the experiments

We tested metaSpectraST on metaproteomics data acquired from 16 fecal samples collected from eight mice at two different time points and compared with the conventional metaproteomics workflow, in which database search against the sample-specific protein sequence database derived from metagenomes were applied. The metagenomes were recovered from 16 metagenomic samples prepared from fecal samples collected from the same set of mice at another two time points prior to the two time points of metaproteomic sampling, respectively (Fig. 1). Metagenomic analysis were also used to characterize the taxonomic composition and genetic potential of the mouse gut microbiomes, which provided an additional insignt into the communities for comparison to the metaproteomics data.

Maternal and co-housing effects are known to be fundamental factors that affect the gut microbiomes in both human and mouse, though such effects may be subtle and unpredictable [53–57]. At the same time, much less is known about the inherent inter-individual heterogeneity even with all known factors well-controlled by the experimentalist. Those unknown factors and related variabilities complicate the design of biological experiments, in particular, in the definition of biological replicates which are necessary for any differential expression analysis. Therefore, a critical prerequisite for gut microbiome research is the ability to rapidly measure the overall similarity and difference between samples, to enable the researcher to identify suitable biological replicates for their experiments. This need is partially met by metagenomic sequencing, which can reveal the taxonomic compositions and genetic potentials of the microbial communities. However, metagenomic sequencing and the subsequent data processing can be costly and time-consuming. Metaproteomics offers a complementary view of the microbial communities, but current workflows are geared towards peptide/protein identification as the first step, which often depends on metagenomic sequencing, and suffers from problems of low sensitivity and ambiguity in protein inference. Therefore, we propose an “inverted” workflow, which quickly assesses inter-sample similarities and differences without peptide/protein identification by spectrum clustering in the first step, and then analyzes the differentiating features between samples in the second step. Such unsupervised learning strategy is useful for detecting outliers and validating biological replicates.

To examine our proposed workflow and explore its utility in microbiome research, we chose 4 littermates from one biological mother and 4 from another biological mother and housed them in different cages with cage mate(s) either from the same or different mothers (Fig. 1) to investigate if maternal or co-housing effects can be detected by our workflow. To illustrate how our method enables one to ask biological questions as a proof of concept, we profiled the communities at two time points shortly after weaning and tested whether we can observe proteome changes that may be associated with the dietary shift from milk to solid food [58]. In addition, we also assessed the effectiveness of metaSpectraST with another metaproteomic dataset of ocean microbiome [59].

### A nonredundant gene set of mouse gut microbiome containing 524,740 genes

We first prepared and sequenced the 16 metagenomic samples and generated an average of about 11 Gb clean reads per sample, each of which was then assembled independently. The number of contigs in one data set ranges from 14,365 to 154,758, and the N50 length ranges from 9653 to 41,799 bp. The rarefaction curves showed all samples approached saturation at the lowest number of reads among data sets, indicating a satisfactory sequencing depth (Supplementary Fig. S1). To create a nonredundant gene set of the 16 metagenomic samples, the predicted ORFs from all of the assembled contigs were clustered and the redundant ones were removed. As a result, a gene set of 524,740 unique genes was created. In comparison, the gene catalog of mouse gut microbiome compiled by Xiao et al. consists of $\sim 2.6$ million unique genes, but such coverage was obtained from a far larger and more diverse cohort of 184 mice [60]. An average of over 80% of the clean reads can be aligned to the nonredundant gene set, suggesting good sequence coverage.

After translating the nucleotide sequences to amino acid sequences, we annotated the nonredundant gene set with the NCBI nonredundant (nr) protein sequence database and the KEGG database. Over 90% of the genes can be functionally annotated by the nr database and nearly 60% can be annotated by the KEGG database. This nonredundant gene set and its annotation was used in the subsequent metaproteomic analysis, in which the derived protein sequence database was used for the identification of peptides and proteins. We also clustered the metagenome-derived protein sequences based on their sequence similarity to reduce the redundancy and complexity of the protein sequence database. The Markov cluster algorithm (MCL) clustered the 534,740 predicted proteins into 156,714 MCL-clustered protein groups, a reduction of about 70% of the size. The members of one MCL-clustered protein group are loosely interpreted as proteins performing the same function across different bacterial species and strains [14, 15, 61].

### 66 MAGs recovered from metagenomic sequences

**Fig. 3:**
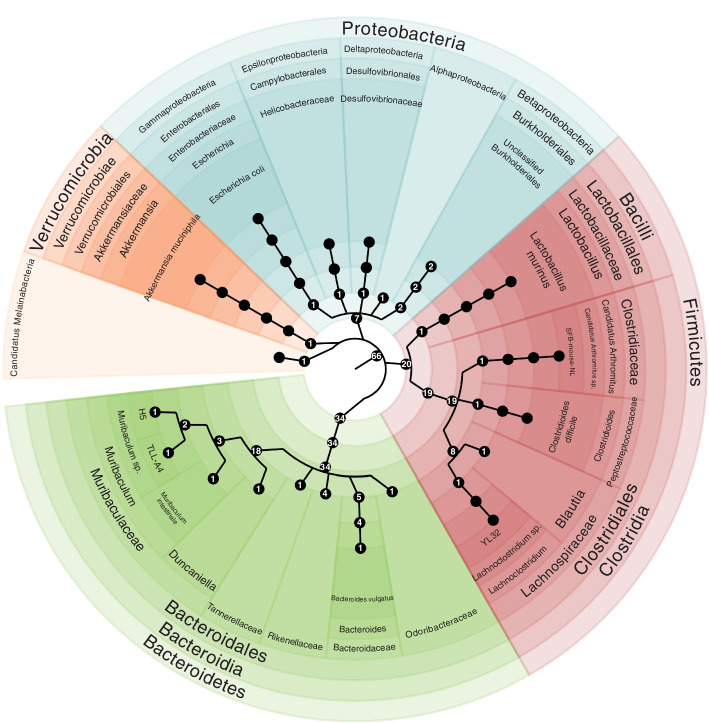
Phylogenetic tree of recovered MAGs. 66 MGAs were recovered from the metagenomic samples. They were from 5 different phyla: Bacteroidetes, Firmicutes, Proteobacteria, Verrucomicrobia, and Candidatus Melainabacteria

By binning the assembled contigs, we successfully recovered 66 bacterial MAGs with high- to medium-quality from the 16 metagenomic samples. Of these 66 MAGs, 2 MAGs (*Odoribacteraceae* and unclassified *Burkholderiales*) were 100% complete, and 58 MAGs (88% of total) were over 90% complete. Most of these MAGs (47 MAGs, 71% of total) were with contamination less than 5% (Supplementary Fig. S2). The 66 MAGs were classified into 5 different phyla: Bacteroidetes, Firmicutes, Proteobacteria, Verrucomicrobia, and Candidatus Melainabacteria (Fig. 3). Bacteroidetes (34 MAGs) and Firmicutes (20 MAGs) comprising 82% of the total MAGs. This taxonomic affiliation of the MAGs was consistent with results from previous studies [60, 62], which also found that Bacteroidetes and Firmicutes were the dominant phyla in the mouse gut microbiome.

**Fig. 4:**
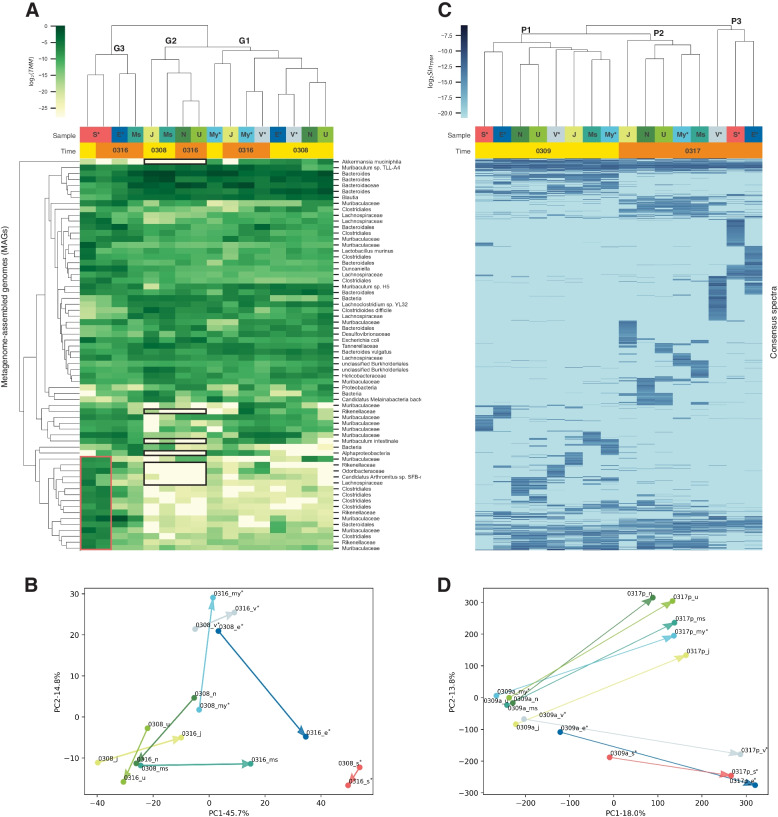
Clustering of mouse gut microbiomes. **A** Heatmap and dendrogram of unsupervised hierarchical clustering of metagenomic samples using relative abundance of MAGs. Rows and columns represent MAGs and samples, respectively. Black boxes highlight MAGs showed significant changes between littermates from different mother mice. Red box highlights MAGs that differentiated gut microbiome of mouse S^*^. **B** PCA of metagenomic samples using relative abundance of MAGs. **C** Heatmap and dendrogram of unsupervised hierarchical clustering of metaproteomic samples using quantitative community profiles (as measured by consensus spectrum $SI_{N}$). Each column corresponds to a sample and each row represents $SI_{N}$ of the consensus spectrum across samples. **D** PCA of metaproteomic samples using consensus spectrum $SI_{N}$. Sample name with and without asterisk indicates littermates of mother mouse 207H and 189C, respectively. Clustering and PCA results based upon consensus spectrum $SI_{N}$ clearly shows microbial changes of the mouse gut microbiome over time; metaproteomic samples collected at the first time point form one cluster (P1) and split into two different clades (P2 and P3) throughout the period of sampling

To explore the microbial composition differences across samples, we estimated the abundance of the 66 MAGs in each metagenomic sample by calculating their standardized read coverage. Unsupervised hierarchical clustering based upon the MAG abundance revealed three major clusters, as shown in Fig. 4A. Metagenomic samples from mice that were not littermates tended to form separate clusters, and they differed from each other predominantly in MAGs from the class of Alphaproteobacteria; the family of *Rikenellaceae, Odoribacteraceae*, and *Lachnospiraceae*; and the species of *Muribaculum intestinale*, *Akkermansia muciniphila*, and *Candidatus Arthromitus sp. SFB-mouse-NL* (Fig. 4A, black boxes). The clustering of mice from the same litter is indicative of the potential maternal effect, in which the microbiome of the mother mouse is assumed to seed the gut microbiome of its offspring, leading to more similarities among mice from the same litter [53, 55, 63].

Notably, the gut microbiomes of mouse N and U, littermates housed together in one cage, showed high similarity from the beginning (right after weaning) to the end (10 days after weaning), while the gut microbiome of mouse S^*^ was distinct from the gut microbiomes of any other mouse throughout the sampling period. The gut microbiome of mouse S^*^ showed extremely high abundance in MAGs from the class of Clostridiales; the family of *Rikenellacea, Odoribacteracea*,* Muribaculaceae*, and *Lachnospiraceae*; and the species of *Candidatus Arthromitus *sp*. SFB-mouse-NL* (Fig. 4A, red box). This is a prime example of wide inter-individual variations in natural microbial communities that cannot be fully controlled by experimental setup. Although littermates S^*^ and E^*^ were housed in the same cage, microbial compositions of their gut microbiomes were found to be quite different even at the beginning, for some unknown reason. Hence, any findings from differential expression analysis would be confounded if we had treated S^*^ and E^*^ as biological replicates. We did not observe clear co-housing effect that can be attributed to inter-individual microbial exchanges over time from the unsupervised hierarchical clustering of the metagenomic samples. We also applied principal component analysis (PCA) to the metagenomic samples, but no clear grouping or separation was found (Fig. 4B). In this study, with a rather small sample size, the data must be interpreted with caution, as the specific findings may not be generalized to other cases. However, the metagenomic results here provided a picture of the gut microbiomes of the mice with respect to taxonomic compositions and genetic potentials and could be used for comparison with the metaproteomic microbial profiles generated by metaSpectraST.

### metaSpectraST detected gut microbiome changes over time

We applied metaSpectraST to all the acquired 377,449 experimental MS/MS spectra from the 16 metaproteomic samples and clustered them as 50,894 spectrum clusters, reducing the data size by 86.5%. 32,413 of the spectrum cluster (63.7% of total) were composed of at least 2 constituent replicate spectra. For each cluster, a consensus spectrum was created by combining all of the constituent replicate spectra of the cluster, generating a set of 50,894 consensus spectra. Spectrum clusters whose consensus spectra were identified as mouse proteins were discarded. We next quantified all consensus spectra in each sample as measured by $SI_{N}$ (*SC* showed a similar result; see Supplementary Figs. S3 and S4) to gain a quantitative metaproteomic profile of the respective microbial community, such that the similarities and differences between samples can be assessed without the need for a protein sequence database. Unsupervised hierarchical clustering based upon the quantitative metaproteomic profiles then revealed that samples collected at different time points formed separate clusters and displayed distinctive patterns of abundance: all samples collected at the first time point (3 days after weaning) were in the same cluster (cluster P1 in Fig. 4C), while samples collected at the second time point (11 days after weaning) formed two different clusters (cluster P2 and P3 in Fig. 4C). The three clusters clearly indicated a divergence of the 16 gut microbiomes over the sampling period, which was further corroborated by the PCA (Fig. 4D).

We believe that these changes in the metaproteomes were likely due to the dietary shift from milk to solid food, which will be further discussed in the context of functional analysis later. Nonetheless, with this expectation, we surmise that these changes in the microbiomes can only be observed by metaproteomics but not by metagenomics, perhaps because the adaptation for the dietary shift was more dependent on changes of protein expression, rather than changes of the taxonomic composition of the microbial community. In other words, the functional profile of the microbial community was adjusted to adapt to the solid food, while its genetic potential remained similar. metaSpectraST offered a new way to detect these possibly dietary-driven microbiome changes over time, giving a complementary picture of the gut microbiome at functional level, which cannot be obtained by metagenomics.

Closer inspection of the three metaproteome clusters showed that samples of cluster P3 were all from the littermates of mother mouse 207H, while samples from littermates of mother mouse 189C were all gathered in cluster P2 except for mouse My^*^. This result suggested the possibility that maternal effect may have an impact on microbiome adjustment to environmental factors, which, again, could not be observed by metagenomics. The exception My^*^ had been expected to show a closely similar metaproteome to mouse V^*^, since they were littermates and housed in the same cage. However, My^*^ was clustered together with mice from a different mother mouse and different cage, which exemplified once more the wide inter-individual heterogeneity of the gut microbiome and demonstrated the ability of metaSpectraST to detect this kind of variabilities and validate biological replicates. But, neither metagenomic analysis nor metaSpectraST uncovered the co-housing effect unambiguously. A larger sample size is probably needed to gain more evidence.

**Fig. 5:**
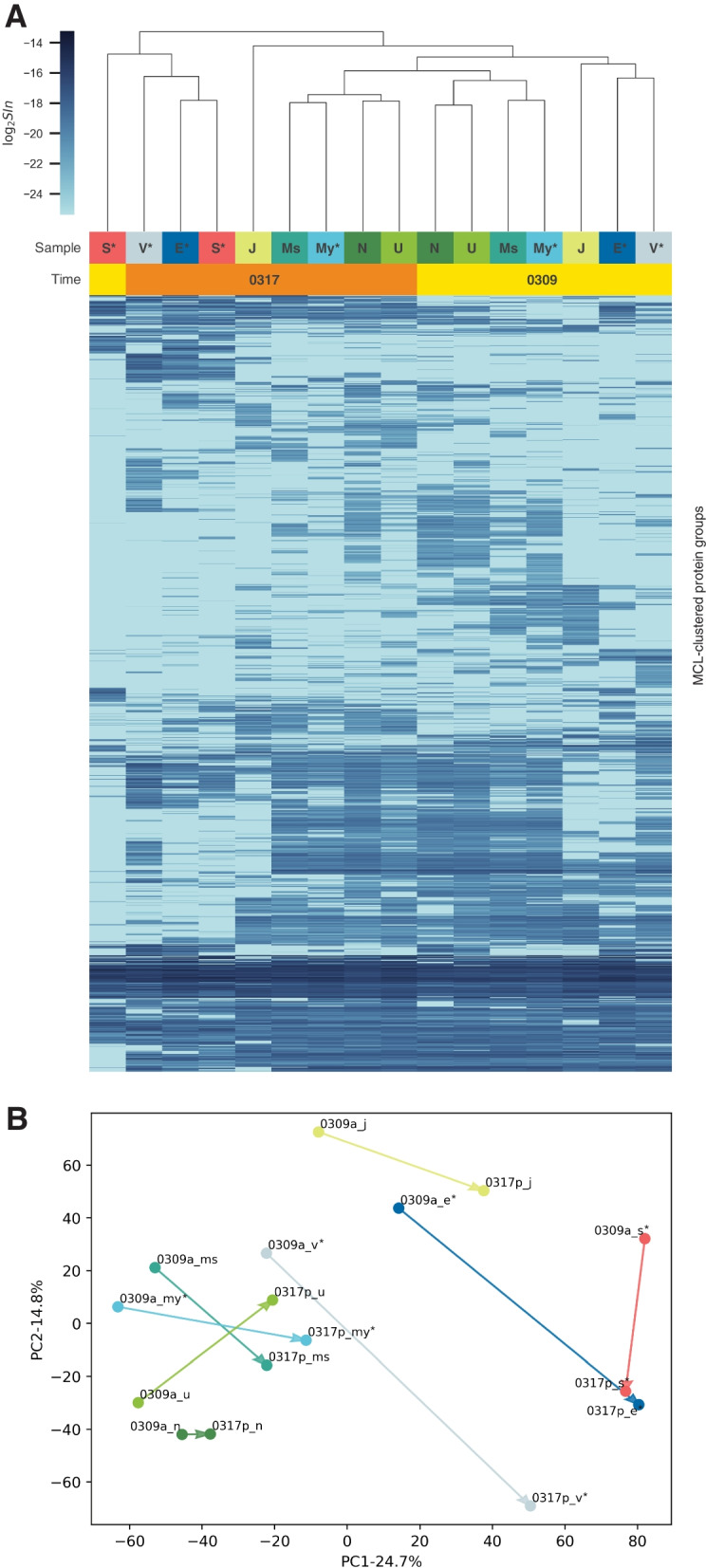
Clustering of metaproteomic samples using $SI_{N}$ of MCL-clustered protein groups. **A** Heatmap and dendrogram of unsupervised hierarchical clustering of metaproteomic samples using $SI_{N}$ of MCL-clustered protein groups. Rows and columns represent MCL-clustered protein groups and samples, respectively. **B** PCA of metaproteomic samples using $SI_{N}$ of MCL-clustered protein groups. Sample name with and without asterisk indicates littermates of mother mouse 207H and 189C, respectively

We further compared metaSpectraST with the traditional metaproteomic workflow, in which database search against the metagenome-derived protein sequence database was applied to identify the spectra to protein(s) (groups). Similarly, we quantified the protein(s) (groups) by $SI_{N}$ as described by Griffin and colleagues [46]. However, more often than not, protein groups inferred from traditional metaproteomics analysis include numerous homologous proteins that have peptide sequences in common, and the list of homologous proteins of a particular protein groups can vary in different samples due to the current protein inference principles of proteomics. The large and varied protein groups make comparative studies across multiple metaproteomic samples complicated and challenging. We modified the published methods from Erickson et al. [16] and Chirania et al. [64] to tackle this problem. In brief, we combined protein groups belonging to the same MCL-clustered protein group (proteins clustered by sequence similarity) and treat each MCL-clustered protein group as a functional entity. The total $SI_{N}$ (sum over all protein groups in one MCL-clustered protein group) is taken to be the level of that biological function commonly performed by proteins in that MCL-clustered protein group. In other words, we compared the $SI_{N}$ of functional groups in each sample. Unsupervised hierarchical clustering and PCA were then applied based upon the $SI_{N}$ values of the MCL-clustered protein groups across samples. This conventional metaproteomics analysis resulted in a less clear classification of the metaproteomic samples and harder to be interpreted (Fig. 5). Therefore we postulated that identifiable spectra only (43.4% of all experimentally observed MS/MS spectra) were inadequate to gain a granular profile of the gut microbiome, as substantial amount of information was contained in those unidentified spectra. Clustering of all experimentally observed MS/MS spectra could preserve the proteomic information of the samples to the maximum, while at the same time making comparison across multiple samples feasible, bypassing the identification of the spectra and protein inference, both of which are time-consuming and error-prone steps particularly in metaproteomics. Similar approaches have been proposed in single-species proteomics, for example, in applications of biological sample fingerprinting [19, 65, 66], but the added complexity of metaproteomic data analysis makes this approach even more valuable.

In addition, to evaluate the effectiveness of metaSpectraST as a metaproteomic workflow, we tested the same workflow with a metaproteomic data set of ocean microbiomes published by Mikan and colleagues in 2020 [59]. The microbiome samples were collected at two different sites from western Arctic Ocean (Bering Strait and Chukchi Sea). Samples were subjected to 10-day shipboard incubation with or without organic material input, referred to as OM group and control group, respectively, to characterize microbial responses of the ocean microbiome to the simulated algal bloom. Without a protein sequence database and peptide identification, metaSpectraST successfully profiled metaproteomes of the ocean microbial communities (Supplementary Fig. S5). Samples collected from Bering Strait and Chukchi Sea formed two different clades at the top hierarchical level, while all biological replicates were clustered together at the lowest hierarchical level displaying the highest similarity to each other. The two initial Bering Strait samples were in the same cluster of Chukchi Sea samples, but after incubation started Bering Strait samples (both OM and control group) diverged to form a new cluster with distinctive proteome changes. This indicated that the incubation process remodeled microbiomes of Bering Strait samples more than that of Chukchi Sea samples. Within each of the two clusters of sampling sites, the OM group was clearly separated from the control group, exhibiting different metaproteome profiles. The organic material input extensively changed the metaproteomes of the samples over time as samples from the OM group showed high dissimilarities at day 6 and day 10 while samples from the control group showed less divergence. These results obtained by metaSpectraST are in accordance with the original findings by metagenomics and conventional metaproteomics analysis, suggesting that metaSpectraST is also effective in other microbiome systems.

### Integrating multiple identification methods with consensus spectra

One important advantage of metaSpectraST is that replicate spectra are grouped and can be identified as a whole, rather than in isolation. First, the consensus spectra can be generated from their constituent replicate spectra, such that the resulting consensus spectra are usually of higher quality than their replicate spectra and hence has a higher chance to be identified. Second, with the assumption that all replicate spectra should stem from the same peptide ion [17–20], one can correct errors by suitably reconciling conflicting identifications among replicates. In this study, multiple identification methods were applied to both the consensus spectra and their constituent replicate spectra to maximize the identification rate. We employed three different identification methods: database search against the metagenome-derived protein database, open modification search against all bacterial protein sequences in UniProt (database independent from metagenomic sequencing and taking more possibilities into consideration), and de novo sequencing. Then, we reconciled any conflicting identification of every cluster among the replicate spectra and the consensus spectrum by majority voting. Thus, to each spectrum cluster, we assigned a consensus peptide sequence, from which the functional or taxonomic information was derived.

At an FDR cutoff of 0.01, 45.1%, 38.6%, and 86.4% of all the experimental MS/MS spectra could be identified by database search, open modification search, and de novo sequencing, respectively (Supplementary Fig. S6). A higher fraction (51.5%) of the consensus spectra could be identified by database search, compared to the experimental spectra, likely because of the high spectral quality of consensus spectra. For each spectrum cluster found by metaSpectraST, a single “consensus peptide” identification was obtained by reconciliation scheme (described in detail in the “Methods” section). Eventually, consensus peptide sequences of 25,667 (50.4% of total), 7236 (14.2% of total), and 10,132 (19.9% of total) of the spectrum clusters were determined by database search, open modification search, and de novo sequencing, respectively. Through the reconciliation scheme, 5062 experimental spectra which could not be identified by database search were rescued by database search of their corresponding consensus spectra, and 20,436 experimental spectra whose peptide sequences assigned by database search disagreed with the majority of sequences of other replicate spectra in the same cluster were finally corrected. We also noticed that after reconciliation there was still 15.4% of the consensus spectra could not be identified to any peptide and therefore would have been neglected in conventional workflow. metaSpectraST preserved those unidentifiable spectra and took advantage of the extra information to classify microbial communities.

In our workflow, we attempted to identify all the experimental spectra, in order to validate our spectrum clustering method. However, in a large study, this may become too time-consuming. In our case, after reconciliation, 98.8% of the consensus peptide sequences determined by database search were identical to the identification of the corresponding consensus spectra. That is to say, if we had only searched the consensus spectrum of each cluster found by metaSpectraST, we could have missed only $\sim$ 1% of the confident identifications, but gained a $\sim$ 7-fold reduction in search time. Therefore, we envision a more efficient workflow for future applications where only the consensus spectra need to be searched.

### Differential functional and taxonomic analysis at the consensus peptide level

**Fig. 6:**
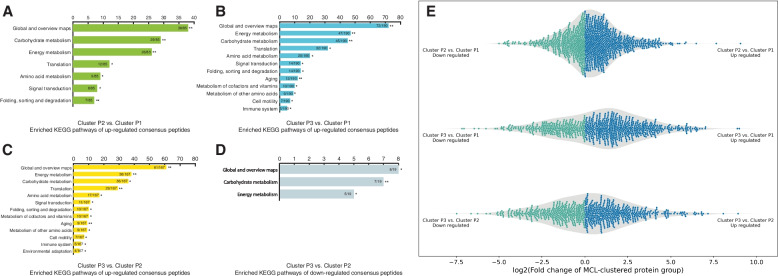
**A**–**D** Enriched KEGG pathways of up- and downregulated consensus peptides between metaproteomic sample clusters. Benjamini-Hochberg Procedure corrected *p*-values are designated as *, *p*-value $<0.05$; **, *p*-value $<0.01$. Fraction on the bar indicates sample frequency over background frequency. **E** Swarm plot showing log2 fold change of MCL-clustered protein groups between three metaproteomic sample clusters (P1, P2, and P3)

To discover whether there were any underlying functional differences between the metaproteomic sample clusters (cluster P1, P2 and P3), we applied Welch’s ANOVA with bootstrapping ($n=10,000$), multiple-testing correction, and post-hoc analysis on the relative abundance of the consensus peptides (as measured by $SI_{N}$) in each cluster. Compared with cluster P1, 188 consensus peptides were significantly upregulated (*p*-value $<0.05$), and 30 were significantly downregulated in cluster P2 (*p*-value $<0.05$) (Supplementary Table S10). The significantly upregulated consensus peptides were enriched in the KEGG pathways of carbohydrate metabolism, energy metabolism, folding and degradation, signal transduction, amino acid metabolism, and translation (with *p*-values ranging from 0.0025 to 0.0175, Fig. 6A and Supplementary Table S11). The downregulated consensus peptides showed no significant pathway enrichment. When comparing cluster P1 with P3, 274 and 9 consensus peptides were significantly up- and downregulated in cluster P3, respectively (*p*-value $<0.05$, Supplementary Table S10). Enrichment analysis showed that the upregulated consensus peptides were enriched in similar KEGG pathways as that of cluster P2 vs. P1 (with *p*-values ranging from 0.0023 to 0.0273, Fig. 6B and Supplementary Table S11), while the downregulated consensus peptides were enriched in glycan biosynthesis and metabolism (*p*-value $=0.025$, Supplementary Table S11). Lastly, we compared cluster P2 and P3 and found 213 significantly overrepresented and 38 significantly underrepresented consensus peptides in cluster P3 (*p*-value $<0.05$, Supplementary Table S10). These differentially represented consensus peptides are enriched in similar pathways to the comparison between cluster P2 vs. P1, and P3 vs. P1 (with *p*-values ranging from 0.0023 to 0.0295, Fig. 6C, D, and Supplementary Table S11). In summary, biological functions related to energy metabolism, carbohydrate metabolism, amino acid metabolism, protein folding and degradation, translation, and signal transduction were remodeled differently in the three metaproteome clusters and might be driven by the dietary shift.

### Differential functional analysis at the MCL-clustered protein group level

We further confirmed the functional differences between the three metaproteomic sample clusters by comparing the relative abundance of the corresponding MCL-clustered protein groups (as functional entities) in each cluster. We plotted the log2 transformed $SI_{N}$ ratios of each MCL-clustered protein groups (only overlapped MCL-clustered protein groups among three clusters) between any two of the three clusters as a swarm plot in Fig. 6E. This swarm plot can be treated as an overall functional comparison between sample clusters, and it displays apparent differences in the log2 fold-change distributions, which confirms the validity of the clustering of the metaproteomic samples. Welch’s ANOVA showed that, compared with cluster P1, 22 and 62 MCL-clustered protein groups were down- and upregulated in cluster P2, respectively (*p*-value $<0.05$). Most of these significantly changed MCL-clustered protein groups were involved in carbohydrate metabolism, lipid metabolism, energy metabolism, amino acid metabolism, nucleotide metabolism, translation, signal transduction, and other biological pathways. Comparison between cluster P1 and P3, and between cluster P2 and P3, resulted in significantly changed MCL-clustered protein groups that were related to the same biological pathways. Therefore, functional analysis at the level of MCL-clustered protein groups suggested functional differences that were in line with the analysis at the consensus peptide level among the three metaproteomic sample clusters.

## Conclusions

In this study, we developed an unsupervised, database-independent workflow, metaSpectraST, as an alternative way to analyze metaproteomic data. metaSpectraST enables rapid microbial community profiling without the need for constructing the protein sequence database or identification of the experimentally observed MS/MS spectra. Instead, metaSpectraST clusters experimental MS/MS spectra solely by their spectral similarity and combines all constituent replicate spectra in one cluster to create the consensus spectrum. Subsequently, the microbial community is quantitatively profiled by counting the number or signal intensity of the constituent replicate spectra of each consensus spectrum in the sample.

We tested the new workflow on 16 mouse gut microbiome samples and compared with the conventional workflow. metaSpectraST successfully detected the possibly dietary-driven mouse gut microbiome changes throughout a period of about 1 week after weaning and managed to separate samples of mice from different mother mice, which might be evidence for the impact of maternal effect. These results demonstrated that metaSpectraST was able to profile the complex gut microbiome and highlight its functional changes. Comparison of metaSpectraST with the traditional workflow indicated that identifiable spectra/peptides alone was not sufficient to profile the microbial community, since spectra that cannot be identified also maintained proteomic information of the community, which can help classify metaproteomic samples. Moreover, correct profiling and classification of ocean microbiome samples demonstrated metaSpectraST’s effectiveness in handling various microbial communities.

Finally, we also showed in our limited study that the proper definition of biological replicates in gut microbiome studies is still an open question for the field. Even though the known variables that affect gut microbiome (e.g., biological mother, cage-mates, food, water, air) had been controlled as best as we could, both metaSpectraST and metagenomic analysis detected the outliers with significant inter-individual variations from the intended biological replicates. We propose that experimentalists should be more cognizant about the unknown factors and random nature of how microbial communities evolve. Despite our best efforts to control the known experimental variables, intended “biological replicates” may not be that similar to begin the experiment with, leading to misleading interpretations of the results. To that end, metaSpectraST can be an invaluable tool for defining and selecting the suitable starting microbial communities for experimentation, thanks to its ability to perform a quick metaproteomic profiling of the microbial communities without any prior knowledge or complicated data processing.

### Supplementary information

**Additional file 1:** **Figure S1.** Rarefaction curve of metagenomic samples. Black vertical line indicates the smallest sample size among all metagenomic samples. All samples are approaching plateau at the smallest sample size. **Figure S2.** (A) Completeness and (B) contamination of MAGs. **Figure S3.** Heatmap and dendrogram of unsupervised hierarchical clustering of metagenomic samples using consensus spectrum *SC*. Each columns corresponds to a sample. Each row represents *SC* of the consensus spectrum across samples. Sample name with and without asterisk indicates littermates of mother mouse 207H and 189C, respectively. Similar to the unsupervised hierarchical clustering of metagenomic samples using consensus spectrum *SI*_N_, samples collected at the first time point form a cluster and diverge over time. **Figure S4.** PCA of metagenomic samples using consensus spectrum *SC*. Sample name with and without asterisk indicates littermates of mother mouse 207H and 189C, respectively. Similar to the unsupervised hierarchical clustering of metagenomic samples using consensus spectrum *SI*_N_, samples collected at the first time point form a cluster and diverge over time. **Figure S5.** Hierarchical clustering of Arctic ocean microbiome samples. **Figure S6.** Venn diagram of identified spectra and sequences. (A) Venn diagram of unique peptide sequences identified from replicate spectra (experimentally observed MS/MS spectra) and consensus spectra. (B) Venn diagram of replicate spectra (experimentally observed MS/MS spectra) that can be identified by database search, open search, and de novo sequencing. **Table S7.** Contigs assembled from each sample. **Table S8.** Genes predicted from each sample. **Table S9.** Identification rate of database search of experimental MS/MS spectra. **Table S10.** Number of up- and down-regulated consensus peptides between sample clusters. **Table S11.** KEGG Enrichment analysis of up- and down-regulated consensus peptides. **Table S12.** Functional annotation of up- and down-regulated consensus peptides.

**Additional file 2.** Table of statistical analysis of significantly changed consensus peptides between sample clusters.

**Additional file 3.** Table of statistical analysis of significantly changed MCL-clustered protein groups between sample clusters.

## Data Availability

The raw sequencing data and genome assemblies of the mice gut microbiome have been deposited in the European Nucleotide Archive under study accession ERP134817. The raw MS/MS data, metagenome-derived protein sequence database and database search results have been deposited to the Mass Spectrometry Interactive Virtual Environment under the dataset accession MSV000089443. metaSpectraST and the user guides are available at https://github.com/bravokid47/metaSpectraST.

## Abbreviations

ORF: Open reading frame
MAG: Metagenome-assembled genome
MCL: Markov cluster algorithm
HCD: Higher-energy collision dissociation
SC: Spectral count
*SI*: Spectral index
*SI*_N_: Normalized spectral index
TMM: Trimmed mean of M values
FDR: False discovery rate
KEGG: Kyoto Encyclopedia of Genes and Genomes
PCA: Principal component analysis
ANOVA: Analysis of variance
