# Supplementary material for: metaSpectraST: an unsupervised and database-independent analysis workflow for metaproteomic MS/MS data using spectrum clustering
Source: Microbiome. 2023 Aug 7;11:176. doi: 10.1186/s40168-023-01602-1 (PMC10405559; doi:10.1186/s40168-023-01602-1)
Supplement: Supplementary file 2 — Additional file 1: Figure S1. Rarefaction curve of metagenomic samples. Black vertical line indicates the smallest sample size among all metagenomic samples. All samples are approaching plateau at the smallest sample size. Figure S2. (A) Completeness and (B) contamination of MAGs. Figure S3. Heatmap and dendrogram of unsupervised hierarchical clustering of metagenomic samples using consensus spectrum SC. Each columns corresponds to a sample. Each row represents SC of the consensus spectrum across samples. Sample name with and without asterisk indicates littermates of mother mouse 207H and 189C, respectively. Similar to the unsupervised hierarchical clustering of metagenomic samples using consensus spectrum SIN, samples collected at the first time point form a cluster and diverge over time. Figure S4. PCA of metagenomic samples using consensus spectrum SC. Sample name with and without asterisk indicates littermates of mother mouse 207H and 189C, respectively. Similar to the unsupervised hierarchical clustering of metagenomic samples using consensus spectrum SIN, samples collected at the first time point form a cluster and diverge over time. Figure S5. Hierarchical clustering of Arctic ocean microbiome samples. Figure S6. Venn diagram of identified spectra and sequences. (A) Venn diagram of unique peptide sequences identified from replicate spectra (experimentally observed MS/MS spectra) and consensus spectra. (B) Venn diagram of replicate spectra (experimentally observed MS/MS spectra) that can be identified by database search, open search, and de novo sequencing. Table S7. Contigs assembled from each sample. Table S8. Genes predicted from each sample. Table S9. Identification rate of database search of experimental MS/MS spectra. Table S10. Number of up- and down-regulated consensus peptides between sample clusters. Table S11. KEGG Enrichment analysis of up- and down-regulated consensus peptides. Table S12. Functional annotation of up- and down-regulated consensus p [file 40168_2023_1602_MOESM1_ESM.pdf]

metaSpectraST: an unsupervised and database-independent analysis workflow for metaproteomic MS/MS data using spectrum clustering

Chunlin Hao, Joshua E. Elias, Patrick K. H. Lee and Henry H. N. Lam

**Supplementary Figure S1**	Rarefaction curve of metagenomic samples
**Supplementary Figure S2**	Completeness and contamination of MAGs
**Supplementary Figure S3**	Hierarchical clustering of metaproteomic samples
**Supplementary Figure S4**	PCA of metaproteomic samples
**Supplementary Figure S5**	Hierarchical clustering of Arctic ocean microbiome samples
**Supplementary Figure S6**	Venn diagram of identified spectra and sequences
**Supplementary Table S7**	Contigs assembled from each sample
**Supplementary Table S8**	Genes predicted from each sample
**Supplementary Table S9**	Identification rate of database search of experimental MS/MS spectra
**Supplementary Table S10**	Number of up- and down-regulated consensus spectra between sample clusters
**Supplementary Table S11**	KEGG Enrichment analysis of up- and down-regulated consensus spectra
**Supplementary Table S12**	Functional annotation of up- and down-regulated consensus spectra

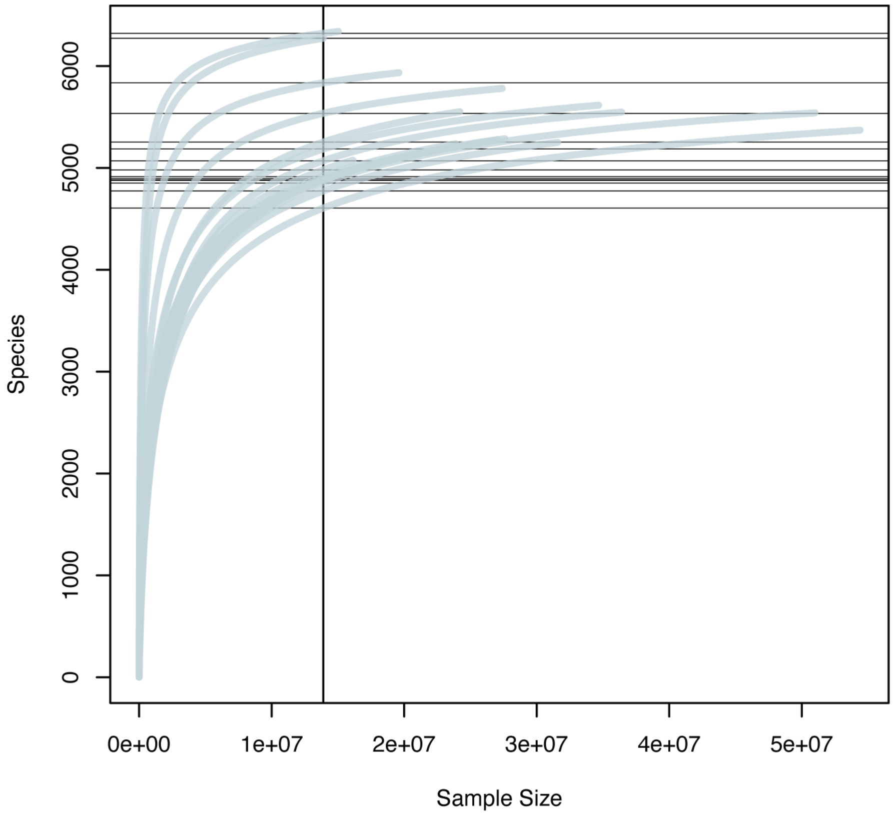

Supplementary Figure S1 | Rarefaction curve of metagenomic samples. Black vertical line indicates the smallest sample size among all metagenomic samples. All samples are approaching plateau at the smallest sample size.

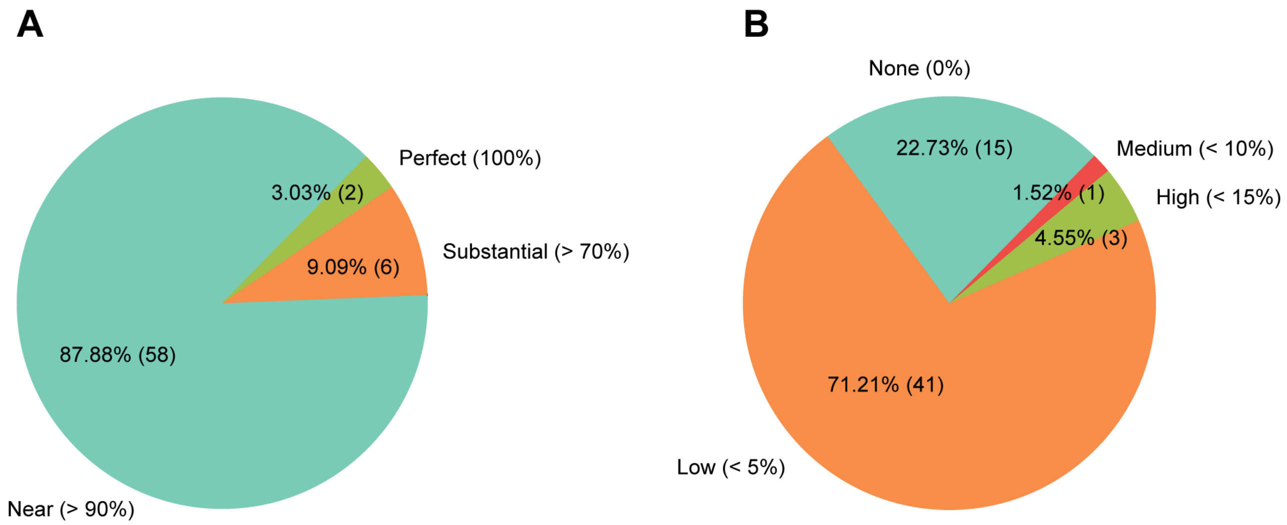

Supplementary Figure S2 | (A) Completeness and (B) contamination of MAGs.

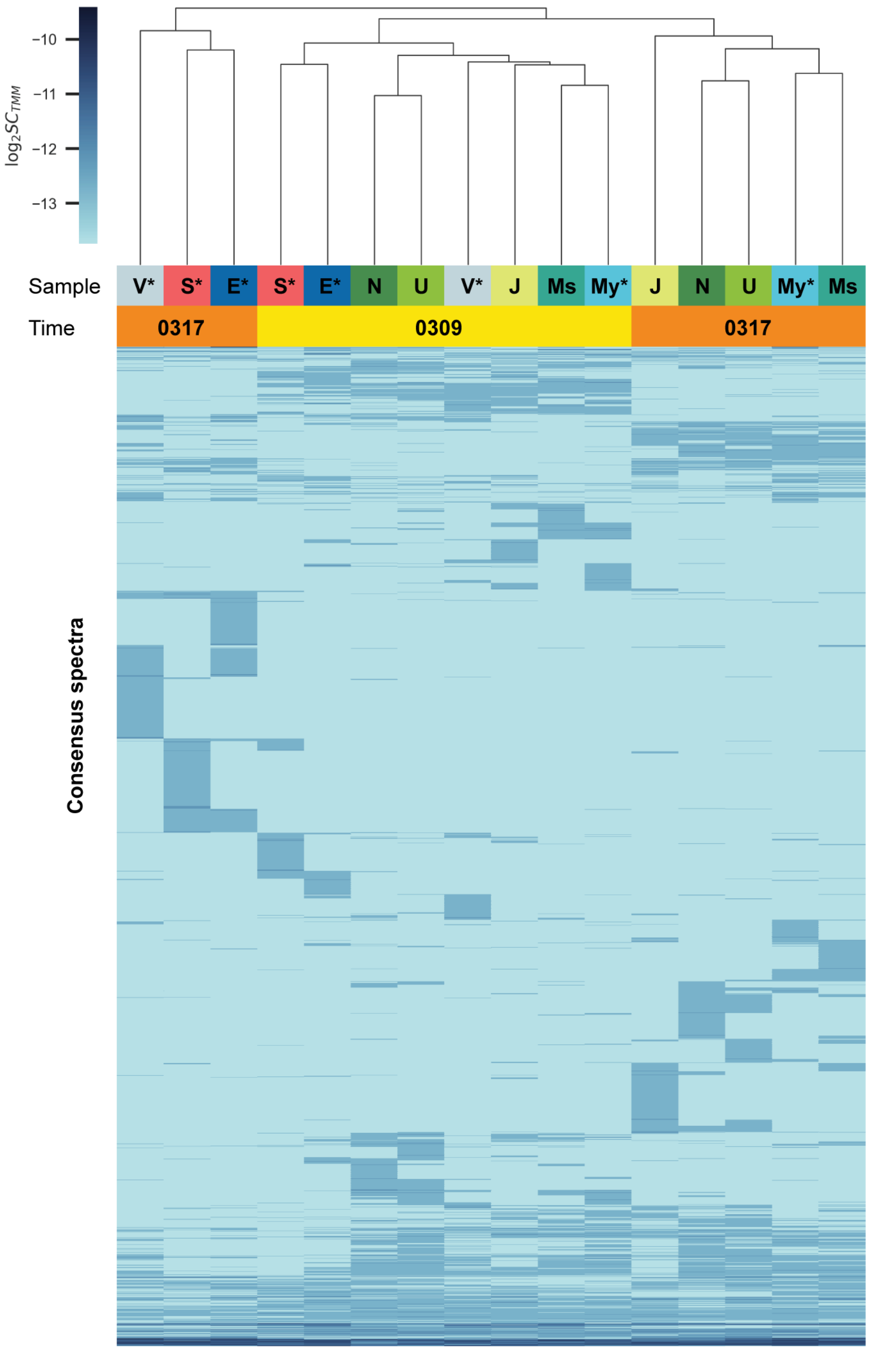

Supplementary Figure S3 | Heatmap and dendrogram of unsupervised hierarchical clustering of metaproteomic samples using consensus spectrum SC. Each columns corresponds to a sample. Each row represents SC of the consensus spectrum across samples. Sample name with and without asterisk indicates littermates of mother mouse 207H and 189C, respectively. Similar to the unsupervised hierarchical clustering of metagenomic samples using consensus spectrum SI_N, samples collected at the first time point form a cluster and diverge over time.

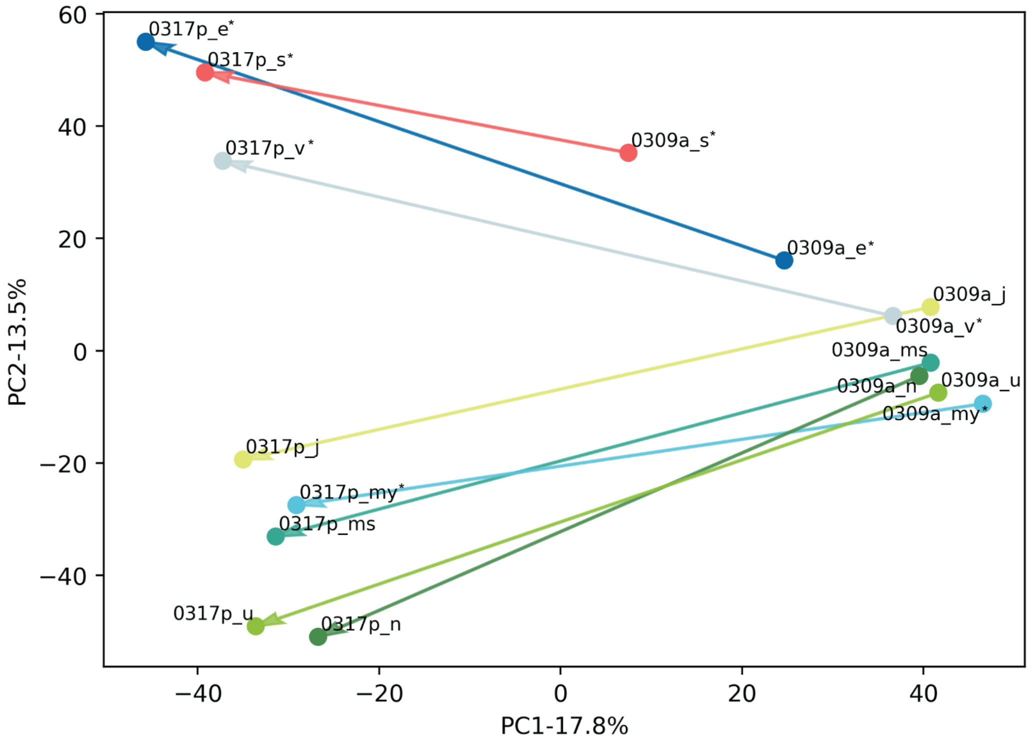

Supplementary Figure S4 | PCA of metaproteomic samples using consensus spectrum SC. Sample name with and without asterisk indicates littermates of mother mouse 207H and 189C, respectively. Similar to the unsupervised hierarchical clustering of metagenomic samples using consensus spectrum SI_N, samples collected at the first time point form a cluster and diverge over time.

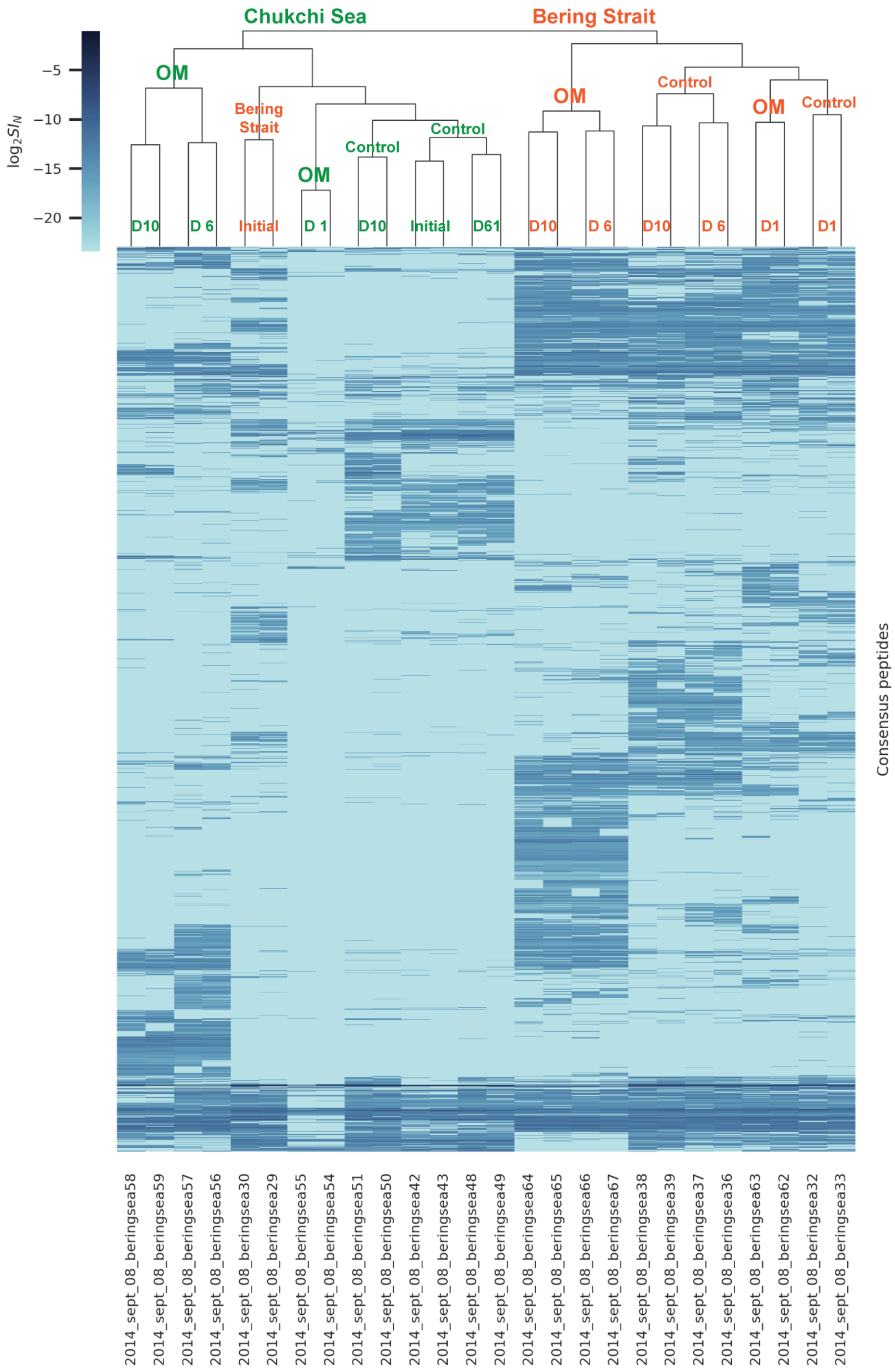

Supplementary Figure S5 | Heatmap and dendrogram of unsupervised hierarchical clustering of Arctic ocean microbiome samples. The data set was published by Mikan, Molly P. et al. previously. Each columns corresponds to a metaproteomic sample. Each row represents SI_N of the consensus spectrum across samples. Annotation on the dendrogram shows the sampling sites of the samples and whether being treated with organic material input.

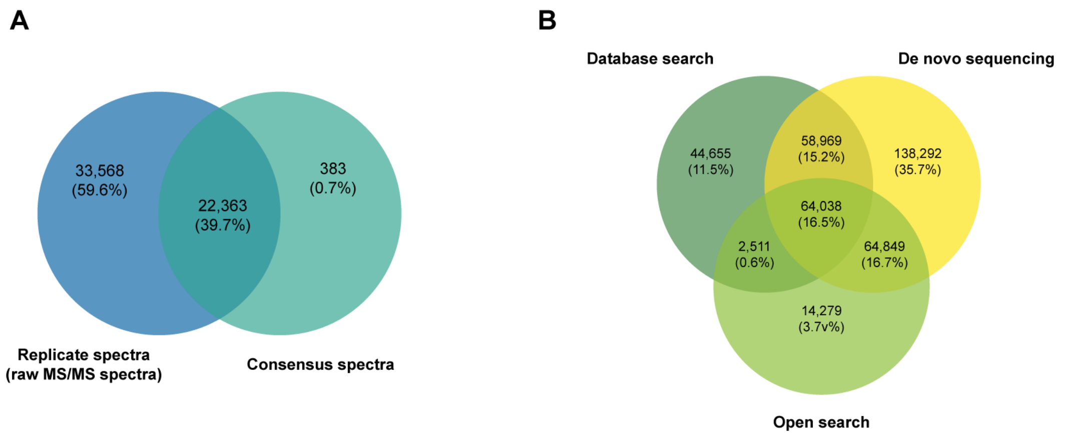

Supplementary Figure S6 | Venn diagram of identified spectra and sequences. (A) Venn diagram of unique peptide sequences identified from replicate spectra (experimentally observed MS/MS spectra) and consensus spectra. (B) Venn diagram of replicate spectra (experimentally observed MS/MS spectra) that can be identified by database search, open search, and *de novo* sequencing.

Supplementary Table S7 I Contigs assembled from each sample.

Sample	# Contigs	Total length (Mb)	N50 (bp)	Avg. length (bp)	Max. length (bp)
0308_E	21,741	98.48	29,228	4,529	310,486
0308_J	14,365	79.81	41,799	5,555	410,450
0308_Ms	20,949	78.68	21,324	3,755	276,938
0308_My	19,819	72.57	25,160	3,661	367,734
0308_N	28,868	82.41	16,020	2,854	222,119
0308_S	96,049	172.94	9,653	1,800	256,614
0308_U	24,173	88.72	26,610	3,670	310,395
0308_V	26,102	92.87	18,420	3,557	367,734
0316_E	53,771	140.77	21,536	2,618	263,650
0316_J	23,970	104.6	31,023	4,363	393,843
0316_Ms	40,626	105.26	15,374	2,590	276,938
0316_My	37,898	104.74	22,528	2,763	367,734
0316_N	30,392	105.72	24,502	3,478	276,938
0316_S	154,758	230.42	4,469	1,488	523,614
0316_U	31,555	109.47	22,099	3,469	263,665
0316_V	27,435	126.0	24,430	4,592	367,734

Supplementary Table S8 l Genes predicted from each sample.

Sample	# Total reads	# Mapped reads	Mapping rate (%)
0308_E	90,343,978	73,817,188	81.71
0308_J	88,451,370	72,015,804	81.42
0308_Ms	83,741,188	68,015,028	81.22
0308_My	55,817,814	45,206,048	80.99
0308_N	83,135,588	67,187,571	80.82
0308_S	84,458,400	63,603,904	75.31
0308_U	97,469,512	79,342,032	81.40
0308_V	79,081,938	63,938,330	80.85
0316_E	151,692,576	123,842,810	81.64
0316_J	172,151,968	143,463,304	83.34
0316_Ms	125,518,328	101,395,723	80.78
0316_My	101,397,856	81,838,408	80.71
0316_N	152,393,462	125,486,576	82.34
0316_S	127,825,340	97,076,577	75.94
0316_U	87,671,582	70,901,986	80.87
0316_V	106,944,716	86,369,907	80.76

Supplementary Table S9 | Identification rate of database search of experimental MS/MS spectra.

Sample	# Spectra searched	# Spectra identified[a]	Identification rate (%)
0309_E	23,477	6,564	28.0
0309_J	23,584	8,133	34.5
0309_Ms	23,429	10,150	43.3
0309_My	24,527	11,660	47.5
0309_N	24,325	11,225	46.1
0309_S	23,749	4,048	17.0
0309_U	24,299	11,618	47.8
0309_V	23,731	8,551	36.0
0317_E	22,713	12,808	56.4
0317_J	24,469	11,800	48.2
0317_Ms	25,012	11,237	44.9
0317_My	24,779	10,538	42.5
0317_N	22,489	14,756	65.6
0317_S	21,657	12,493	57.7
0317_U	22,609	13,061	57.8
0317_V	22,600	13,124	58.1

[a]PeptideProphet estimated FDR = 0.01.

Supplementary Table S10 | Number of up- and down-regulated consensus peptides between sample clusters.

Comparison	# Up-regulated				# Down-regulated			
	Database search[a]	Open search[b]	*de novo* sequencing[c]	N/A[d]	database search	Open search	*de novo* sequencing	N/A
P2 vs. P1	85	28	53	22	7	6	17	0
P3 vs. P1	190	23	44	17	2	2	5	0
P3 vs. P2	167	11	21	14	19	5	9	5

Welch's ANOVA with bootstrapping ($n = 10,000$) and Benjamini-Hochberg procedure were used to determine significantly changed consensus peptides (FDR = 0.05); Games-Howell test was used for post-hoc analysis with a p-value of 0.05.
[a]Number of consensus spectra that finally determined by database search (after reconciliation).
[b]Number of consensus spectra that finally determined by open search (after reconciliation).
[c]Number of consensus spectra that finally determined by *de novo* sequencing (after reconciliation).
[d]Number of consensus spectra that cannot be identified.

Supplementary Table S11 | KEGG Enrichment analysis of up- and down-regulated consensus peptides.

KEGG pathway (level 2)	# Hits	p-unc[a]	BH[b]	Max. FC[c]	Min. FC
**P2 vs. P1, up-regulated consensus spectra**					
Energy metabolism	26	2.37e-12	0.0025	691.22	15.39
Global and overview maps	38	2.29e-11	0.0050	691.22	15.39
Carbohydrate metabolism	29	2.10e-09	0.0075	691.22	15.39
Folding, sorting and degradation	7	5.02e-04	0.0100	193.07	21.08
Signal transduction	8	9.87e-03	0.0125	464.97	17.72
Amino acid metabolism	9	9.47e-03	0.0150	94.42	16.47
Translation	12	1.32e-02	0.0175	140.26	9.67
**P2 vs. P1, down-regulated consensus spectra, no significant enrichment**					
**P3 vs. P1, up-regulated consensus spectra**					
Energy metabolism	47	9.78e-16	0.0023	652.58	20.74
Global and overview maps	72	3.94e-13	0.0045	652.58	13.61
Aging	12	1.13e-07	0.0068	253.35	23.49
Carbohydrate metabolism	45	9.86e-07	0.0091	652.58	13.61
Translation	32	3.73e-06	0.0114	526.39	12.13
Folding, sorting and degradation	14	6.02e-06	0.0136	253.35	23.49
Metabolism of other amino acids	9	1.06e-04	0.0159	184.31	17.58
Signal transduction	14	3.63e-04	0.0182	359.04	24.45
Amino acid metabolism	20	4.48e-04	0.0205	235.57	14.90
Metabolism of cofactors and vitamins	10	1.04e-03	0.0227	292.64	14.90
Cell motility	7	1.37e-02	0.0250	359.04	53.48
Immune system	5	1.43e-02	0.0273	265.40	53.48
**P3 vs. P1, down-regulated consensus spectra**					
Glycan biosynthesis and metabolism	1	2.23e-02	0.0250	0.01	0.01
**P3 vs. P2, up-regulated consensus spectra**					
Energy metabolism	38	8.43e-12	0.0023	457.62	3.17
Global and overview maps	61	1.57e-10	0.0045	457.62	3.17
Translation	29	4.19e-06	0.0068	903.26	14.9
Aging	9	1.60e-05	0.0091	268.54	35.95
Metabolism of other amino acids	9	3.69e-05	0.0114	282.09	26.91
Carbohydrate metabolism	36	1.30e-04	0.0136	457.62	19.7
Metabolism of cofactors and vitamins	10	3.58e-04	0.0159	163.14	19.96
Folding, sorting and degradation	10	6.68e-04	0.0182	268.54	35.95
Amino acid metabolism	17	1.59e-03	0.0205	284.64	3.17
Signal transduction	11	3.51e-03	0.0227	784.16	20.55
Cell motility	7	6.74e-03	0.0250	784.16	81.86
Immune system	5	8.27e-03	0.0273	405.91	81.86
Environmental adaptation	4	2.87e-02	0.0295	264.66	81.86
**P3 vs. P2, down-regulated consensus spectra**					
Carbohydrate metabolism	7	1.20e-03	0.0050	0.01	0.61
Global and overview maps	8	2.49e-03	0.0100	0.01	0.61
Energy metabolism	5	3.83e-03	0.0150	0.01	0.61

[a]Two-tailed p-value of Fisher's exact test.
[b]Benjamini-Hochberg procedure corrected p-value.
[c]FC: fold-change.

Supplementary Table S12 | Functional annotation of up- and down-regulated consensus peptides.

Consensus spectrum	Sequence	NR annotation[a]	MCL-clustered protein group
**P3 vs. P2, up-regulated consensus spectra**			
0317p_v_25209/2	SGVLGISGVSSDFR	WP_034373626.1 I Acetate kinase [Helicobacter sp. MIT 05-5293]	Acetate kinase
0317p_v_25261/2	VDFNVPLDENGNITDDTR	CCX48228.1 I Phosphoglycerate kinase [Bacteroides sp. CAG:927]	Phosphoglycerate kinase
0317p_s_29363/2	TILWNGPAGVFEFDNFTAGSR	CCX48228.1 I Phosphoglycerate kinase [Bacteroides sp. CAG:927]	Phosphoglycerate kinase
0317p_e_14506/3	HFGAQGIGLCR	CCX47821.1 I Pyruvate phosphate dikinase [Bacteroides sp. CAG:927]	Pyruvate, phosphate dikinase
0309a_e_15134/2	TEHMFFEGDR	CCX47821.1 I Pyruvate phosphate dikinase [Bacteroides sp. CAG:927]	Pyruvate, phosphate dikinase
0317p_s_21155/2	WAALQGISEEER	CCX47821.1 I Pyruvate phosphate dikinase [Bacteroides sp. CAG:927]	Pyruvate, phosphate dikinase
0317p_v_25055/3	ANDMGVICGVTTNPSLIAK	WP_007862230.1 I MULTISPECIES: fructose-6-phosphate aldolase [Clostridiales]	Fructose-6-phosphate aldolase
0317p_e_30043/3	GILGYTEDAVVSSDFLGDPR	WP_068961616.1 I MULTISPECIES: type I glyceraldehyde-3-phosphate dehydrogenase [Bacteroidales]	Type I glyceraldehyde-3-phosphate dehydrogenase
0317p_e_19414/3	DMQDMEFTVEHGK	WP_016316361.1 I Pyruvate, phosphate dikinase [Anaerotruncus sp. G3(2012)]	Pyruvate, phosphate dikinase
0317p_s_27263/3	LNTHIGIGIPWDLDR	WP_022741572.1 I Glucose-1-phosphate adenylyltransferase [Lachnospiraceae bacterium COE1]	Glucose-1-phosphate adenylyltransferase
0317p_v_31541/2	QMFGAEGYEVVAINDLTSPK	WP_016290705.1 I Type I glyceraldehyde-3-phosphate dehydrogenase [Lachnospiraceae bacterium 28-4]	Type I glyceraldehyde-3-phosphate dehydrogenase
0317p_v_23979/2	IGMLTSGGDCQALNAAMR	CDF06585.1 I 6-phosphofructokinase 1 [Firmicutes bacterium CAG:95]	6-phosphofructokinase
0317p_v_19793/2	YVQGFPEYGPER	WP_016302159.1 I 2-ketoisovalerate family 2-oxoacid:acceptor oxidoreductase subunit gamma [Lachnospiraceae bacterium COE1]	2-ketoisovalerate family 2-oxoacid:acceptor oxidoreductase subunit gamma
0317p_e_24554/2	FWGLGGDGTVGANK	WP_087261578.1 I Pyruvate:ferredoxin (flavodoxin) oxidoreductase [Flavonifractor sp. An92]	Pyruvate:ferredoxin (flavodoxin) oxidoreductase
0317p_s_22618/2	VLLFNDGAVTGR	WP_016290431.1 I Phosphoenolpyruvate carboxykinase (ATP) [Lachnospiraceae bacterium 28-4]	Phosphoenolpyruvate carboxykinase (ATP)
0317p_e_17859/2	LETSPEDITGMK	WP_007887525.1 I Pyruvate, phosphate dikinase [Roseburia inulinivorans]	Pyruvate, phosphate dikinase
0317p_v_25706/3	DATPMFVCGVNFDKYEK	WP_094384019.1 I Type I glyceraldehyde-3-phosphate dehydrogenase [Prevotella sp. P2-180]	Type I glyceraldehyde-3-phosphate dehydrogenase
0317p_e_27923/3	NLDTLLHPQFDAAALK	WP_016282501.1 I Pyruvate, phosphate dikinase [Lachnospiraceae bacterium A4]	Pyruvate, phosphate dikinase
0317p_v_23927/3	LKGSALASVMGATLATK	WP_009757376.1 I Phosphoenolpyruvate carboxykinase (ATP) [Lachnospiraceae bacterium 2_1_46FAA]	Phosphoenolpyruvate carboxykinase (ATP)
0317p_v_20691/2	CGSIGYGIATAYAK	WP_016282402.1 I SDR family oxidoreductase [Lachnospiraceae bacterium A4]	SDR family oxidoreductase
0317p_s_23094/3	GSQFKQPLLEFSGACAGCGETPYAK	WP_026347346.1 I Pyruvate:ferredoxin (flavodoxin) oxidoreductase [Eubacterium]	Pyruvate:ferredoxin (flavodoxin) oxidoreductase
0317p_s_26343/3	ASNAPELFAKPDIDGGLIGGASLK	WP_104369505.1 I Unknown	Triose-phosphate isomerase
0317p_e_25686/2	VMVIPTNEELAIAR	WP_103239817.1 I Unknown	Acetate kinase
0317p_e_26534/2	MTIQELVNNAR	WP_004036483.1 I Hypothetical protein [Clostridium sp. ASF356]	Hypothetical protein
0317p_s_10421/2	AQQTGEIETIK	WP_004033200.1 I Gfo/Idh/MocA family oxidoreductase [Clostridium sp. ASF356]	Gfo/Idh/MocA family oxidoreductase

Continued on next page

Continuation of Supplementary Table S11

Consensus spectrum	Sequence	NR annotation[a]	MCL-clustered protein group
0317p_e_22322/2	EVSWCPSYGPEMR	WP_004034236.1 I Hypothetical protein [Clostridium sp. ASF356]	2-oxoglutarate ferredoxin oxidoreductase subunit gamma
0317p_e_19739/2	MCPDSAITVEKID	WP_004034245.1 I Ferredoxin family protein [Clostridium sp. ASF356]	Ferredoxin
0317p_e_28313/2	VVGVAPGILEATGLR	WP_004038148.1 I NAD(P)-dependent oxidoreductase [Clostridium sp. ASF356]	NAD(P)-dependent oxidoreductase
0317p_v_28524/2	FLIPVDTVITK	WP_072852263.1 I Phosphoglycerate kinase [Clostridium]	Phosphoglycerate kinase
0317p_e_22218/3	KLGDPEDPLLVSVR	WP_072850481.1 I Pyruvate, phosphate dikinase [Clostridium]	Pyruvate, phosphate dikinase
0317p_v_19992/2	IVQMNYDAIDAGAK	WP_016294518.1 I Pyruvate:ferredoxin (flavodoxin) oxidoreductase [Lachnospiraceae bacterium M18-1]	Pyruvate:ferredoxin (flavodoxin) oxidoreductase
0317p_e_26611/2	VDILEAYQVGGAAVK	WP_087150025.1 I 6-phosphofructokinase [Lachnoclostridium sp. An298]	6-phosphofructokinase
0317p_v_21823/2	EVTVFSCMDPK	WP_088108212.1 I Type I glyceraldehyde-3-phosphate dehydrogenase [Tyzzerella sp. An114]	Type I glyceraldehyde-3-phosphate dehydrogenase
0317p_e_23408/2	IGAFGIPYSR	WP_066090082.1 I Acyl-CoA dehydrogenase [Clostridium]	Acyl-CoA dehydrogenase
0317p_v_28915/3	IGIGAQSVGIAQGAIDEAMK	WP_066090082.1 I Acyl-CoA dehydrogenase [Clostridium]	Acyl-CoA dehydrogenase
0317p_s_11850/2	AASNCAVTYENVR	WP_066090082.1 I Acyl-CoA dehydrogenase [Clostridium]	Acyl-CoA dehydrogenase
**P3 vs. P2, down-regulated consensus spectra**			
0317p_n_18371/3	GMAENGDVFFQHR	OKY85570.1 I Pyruvate:ferredoxin (flavodoxin) oxidoreductase [Bacteroidales bacterium 52_46]	Pyruvate:ferredoxin (flavodoxin) oxidoreductase
0317p_e_17569/3	FAWDSYRR	CCX47821.1 I Pyruvate phosphate dikinase [Bacteroides sp. CAG:927]	Pyruvate, phosphate dikinase
0317p_j_28427/2	ELHTFLNNELPEIDWR	WP_107031240.1I Unknown	Transketolase
0317p_j_17748/3	RFEGLNPVHTPGVLVK	WP_005826585.1 I MULTISPECIES: L-ribulose-5-phosphate 4-epimerase [Bacteria]	L-ribulose-5-phosphate 4-epimerase
0317p_j_27131/3	IVEFELNDAEKELFHK	OKY86473.1 I Malate dehydrogenase [Bacteroidales bacterium 52_46]	Malate dehydrogenase
0317p_n_24072/2	ELTTISLSPEAISR	WP_094582497.1 I Phospho-sugar mutase [Parabacteroides sp. CT06]	Phospho-sugar mutase
0317p_ms_27340/3	ELLKDSLDQIFEHK	WP_107032898.1 I Unknown	L-rhamnose isomerase

[a]Annotated by NCBI non-redundant protein database
